# Influence of curcumin supplementation on metabolic and lipid parameters of people living with HIV/AIDS: a randomized controlled trial

**DOI:** 10.1186/s12906-019-2620-7

**Published:** 2019-08-06

**Authors:** Tatiane Andreza Lima Silva, Danielle Coutinho Medeiros, Gidyenne Christine Bandeira Silva Medeiros, Rafaela Catherine Silva Cunha Medeiros, Juliany de Souza Araújo, Jason Azevedo Medeiros, Marcela Abbott Galvao Ururahy, Ronaldo Vagner Thomatieli Santos, Radames Maciel Vitor Medeiros, Lucia Leite-Lais, Paulo Moreira Silva Dantas

**Affiliations:** 1 Postgraduate Program in Health Science, Federal University of Rio Grande do Norte, Av. Nilo Peçanha 620, Petropolis, Natal, RN 59012-300 Brazil; 2 Department of Physical Education, Federal University of Rio Grande do Norte, Campus Universitario, Lagoa Nova, Natal, RN 59078-900 Brazil; 3 Department of Nutrition, Federal University of Rio Grande do Norte, Campus Universitario, Lagoa Nova, Natal, RN 59078-900 Brazil; 4 Department of Clinical and Toxicological Analyses, Centro de Ciencias da Saude, Petropolis, Natal, RN 59012-300 Brazil; 5 Bioscience Department, Federal University of Sao Paulo, UNIFESP, Avenida Ana Costa, 96, Vila Matias, Santos, SP 11060-001 Brazil; 6 Centro Universitário do Rio Grande do Norte, UNIRN, Rua Prefeita Eliane Barros, 2000, Tirol, Natal, RN 59014-545 Brazil

**Keywords:** Curcumin, Dyslipidemia, HIV/Aids

## Abstract

**Background:**

Scientific studies have shown that the potential therapeutic efficacy of curcumin in several diseases is due to its potent antioxidant and anti-inflammatory properties. Consequently, curcumin supplementation seems to be a valuable alternative for HIV-infected individuals. The aim of this study is to evaluate the influence of curcumin supplementation on substrate oxidation at rest, body composition, and the lipid profile of physically active people living with HIV/AIDS under antiretroviral therapy.

**Methods:**

This double-blind, crossover, randomized clinical trial was comprised of 20 subjects divided into experimental (EG) and control (CG) groups, receiving 1000 mg curcumin/day and placebo, respectively, during a 30-day period. Substrate oxidation at rest was assessed by indirect calorimetry, body composition was measured by dual-energy x-ray absorptiometry, and the lipid profile was evaluated by blood tests. Data analysis was performed by independent samples and paired t-tests to compare the differences between groups and times. A *p*-value < 0.05 was accepted as significant.

**Results:**

There were no differences between groups regarding substrate oxidation at rest or body composition. However, serum triglyceride levels were increased after curcumin supplementation (182 vs. 219 mg/dL; *p* = 0.004).

**Conclusion:**

Curcumin supplementation promoted the elevation of serum triglyceride levels in HIV-infected subjects. Further studies with a larger sample cohort, different curcumin doses, and longer intervention times are needed to validate current observations. In addition, the influence of physical activity, dietary intake, and genetic polymorphisms must be considered in future studies to better understand the impact of curcumin supplementation on the lipid profile of people living with HIV/AIDS under antiretroviral therapy.

**Electronic supplementary material:**

The online version of this article (10.1186/s12906-019-2620-7) contains supplementary material, which is available to authorized users.

## Background

Antiretroviral therapy (ART) is able to dramatically increase the survival and quality of life in people living with HIV/AIDS. However, these advantages are often associated with metabolic alterations and risk of cardiovascular diseases, which are difficult to manage [1].

Strategies to reduce cardiovascular risk should be prioritized in the health care of people using ART. In most cases, lifestyle changes are not enough to reverse the atherogenic dyslipidemia observed in this population. Besides healthy diet, regular physical activity, smoking cessation and alcohol abstinence, the use of conventional lipid lowering drugs are needed [1–3]. Nevertheless, prescription of lipid lowering drugs in this population requires strict clinical monitoring due to its possible pharmacokinetic interactions with antiretroviral drugs [4]. Moreover, the concomitant use of fibrates and statins increases the risk of skeletal muscle toxicity and rhabdomyolysis [5–7].

Bioactive compounds naturally present in foods, which are able to modulate serum lipid levels in favor of a less atherogenic profile, may have a special role in this population. Curcumin, a polyphenol extracted from *Curcuma longa L*. commonly known as turmeric, has been highlighted due to its effects on many biological pathways and it is considered safe [8]. Despite the challenges of curcumin action due to its low solubility and poor bioavailability, its antiviral, anti-inflammatory, and antioxidant properties have pointed to curcumin as a promising bioactive compound with a positive effect on the treatment of HIV infection and AIDS [9, 10], inflammatory diseases [11], cancer [12], diabetes [13], and metabolic syndrome [14]. Curcumin acts on molecular targets associated with intestinal cholesterol absorption and regulation of low-density lipoprotein (LDL) receptors and it may be helpful to improve the lipid profile [15].

The inflammatory process and oxidative stress caused by the HIV virus and ART lead to metabolic alterations, such as dyslipidemia [16], lipodystrophy [17], and insulin resistance [18], which increase the risk of cardiovascular events. Therefore, curcumin supplementation as a non-drug strategy seems to be a valuable alternative for HIV-infected people under ART. Thus, the aim of this study was to evaluate the effect of curcumin supplementation on substrate oxidation at rest, body composition, and lipid profile of people living with HIV/AIDS and under ART.

## Methods

### Study design, ethical aspects, and supplementation

This study was a double-blind, crossover, randomized clinical trial, conducted according to the Declaration of Helsinki, between September and December 2017. It was reviewed and approved by the Human Research Ethics Committee of the Federal University of Rio Grande do Norte, Brazil (CAAE 42950214.7.0000.5537), and was registered in Clinical Trials (NCT03141918), available at https://clinicaltrials.gov/ct2/show/NCT03141918. Written consent was obtained from all participants. Subjects were randomized 1:1 to the experimental group (EG) or control group (CG), supplemented with curcumin or placebo, respectively.

The randomization was performed by an independent researcher using the Research Randomizer, a free online tool. He kept the original random allocation sequences in an inaccessible location and worked with a copy. Participants received identification codes which were revealed at the end of the study. The curcumin or placebo supplementation was distributed by the independent researcher and both capsules were similar in color and size. The study was double blind, meaning that neither participants nor investigators responsible for conducting the assessments were aware of the allocated arm until the statistical analysis was performed.

**Fig. 1:**
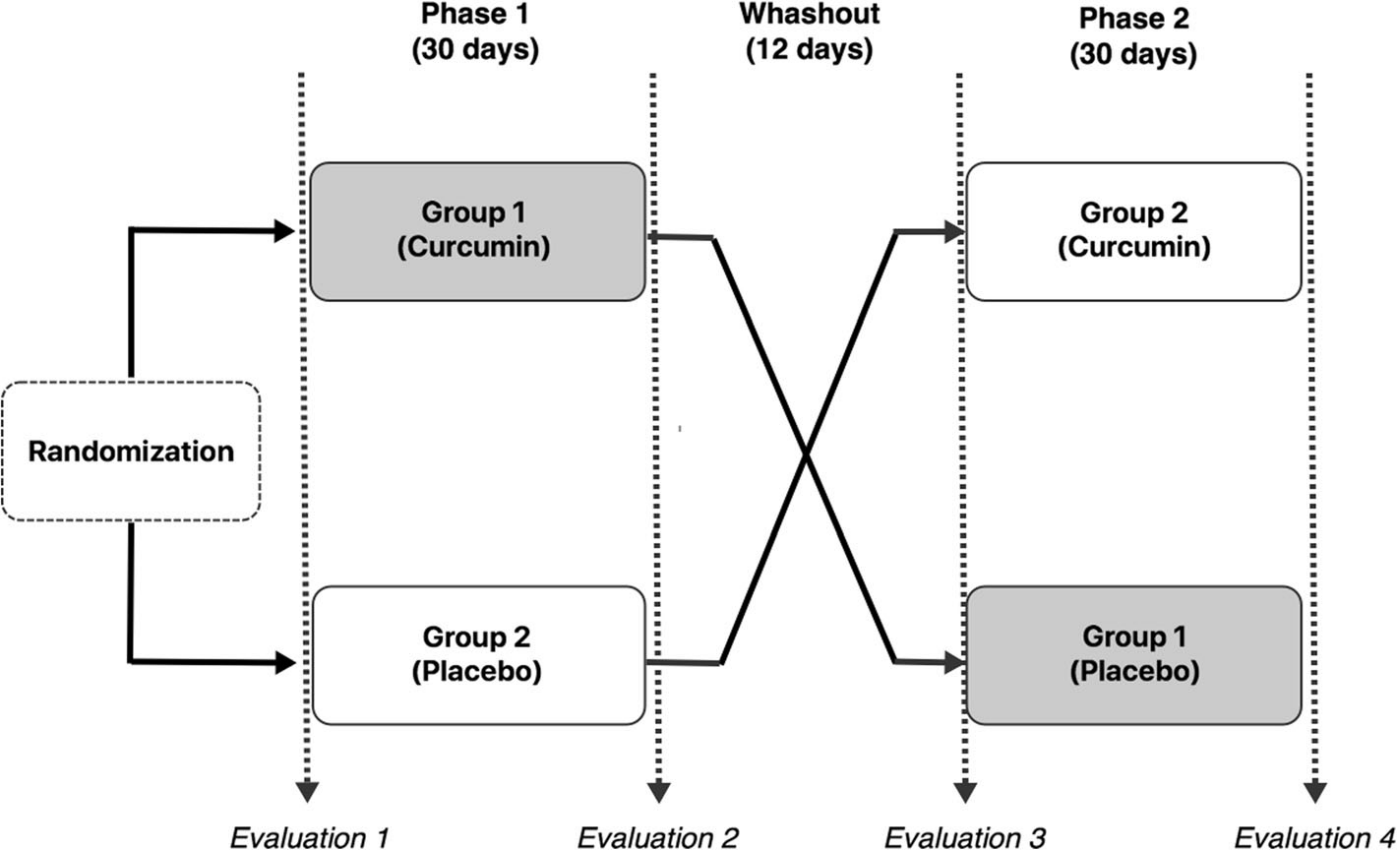
Study Design. Legend: This figure shows the formulation of the trial, including the randomization process, phase 1, washout, and phase 2 periods. Also, it shows the groups 1 and 2 as experimental group (EG) and control group (CG), respectively, and their cross-over during the follow up. In addition, four reference points for evaluations 1–4 are pointed out. Each evaluation was comprised by full assessment, including anamnesis, 24-h dietary recall, indirect calorimetry, dual-energy X-ray absorptiometry, blood collection, and biochemical analysis

The EG and the CG received 1000 mg curcumin/day for 30 days (BioMor® Curcumin) and 1000 mg microcrystalline cellulose (placebo), respectively, divided into two capsules of 500 mg each, which were taken with water two hours after breakfast and two hours after lunch. After 30 days of supplementation, a washout period of 12 days was implemented. For logistical issues, this period was longer than the washout period recommended in clinical trials with curcumin supplementation, which is 7 days [19]. Afterwards, the groups were switched to follow the crossover design. Full assessments were performed at four different intervals (Fig. 1).

### Participants

This study was carried out with people living with HIV/AIDS of both sexes, members of the Viver+ program, offered by the Federal University of Rio Grande do Norte (UFRN). This program offers nutritional advice and exercise guidance to this population. The sampling method adopted was non-probability for convenience. The data were collected at the Physical Education Department at UFRN in Natal, Brazil.

The participants of this study were previously enrolled in a 6-month exercise plan, prescribed according to the American College of Sports Medicine [20]. The exercise plan consisted of a combination of aerobic and resistance training, 3 times/week on nonconsecutive days, with moderate intensity, for 50 min (10 min of aerobic training and 40 min of resistance training). At baseline, participants presented a mean VO2 peak of 33.7 mL/kg/min and were physically active according to the International Physical Activity Questionnaire classification [21]. Throughout the study, participants were advised not to change their lifestyle, including diet and physical activity. Thus, during the study all participants continued to follow the exercise plan, which was monitored by Physical Education professionals through the Borg [22] and OMNI-RES [23] subjective perception of exertion scales for aerobic and resistance training, respectively.

Inclusion criteria were subjects over 18 years of age who had been using ART for at least 18 months, sufficient time to be vulnerable to the side effects of the treatment. Exclusion criteria to avoid bias were subjects using *Curcuma longa L.*, taking curcumin or thermogenic supplements, with acute infection, pregnant individuals, and exercise training attendance below 75%. Information was registered on the evaluation form (Additional file 1).

### Study procedures

#### Anamnesis

A semi-structure questionnaire was developed for this study to collect personal, lifestyle, and clinical data, including the time of HIV diagnosis, time taking ART, combination of medications used, and the presence of comorbidities (see Additional file 1).

#### Dietary intake

Dietary intake was evaluated as a control variable, since the participants were advised not to change their eating habits during the study. For this, food intake was investigated by trained registered dietitians through 24-h recalls, according to the literature recommendations [24]. Four 24-h recalls were collected for each patient during evaluations 1–4 (Fig. 1). A picture book with food portions and measurements was used to estimate portion sizes. Dietary energy, macronutrients, cholesterol, and dietary fiber were measured applying Diet Easy® software. Food items that were not found in the software’s databank were added based on the nutrition facts labels.

In addition, participants filled out a questionnaire, called Food Consumption Markers, developed by the Brazilian Ministry of Health [25]. The purpose of this tool was to check the weekly frequency of consumption of 10 food groups organized according to their level of food processing as well as sources of specific nutrients, such as sugar, saturated fat, and fiber. Groups 1 to 5 comprise unprocessed or minimally processed foods, while groups 6 to 10 comprise processed or ultra-processed foods. The analysis of the Food Consumption Markers collected was performed based on the following equation: Score = 1 / 7 x consumption frequency, adapted from Fornés et al. [26]. The results were expressed as percentages.

#### Substrate oxidation at rest

Evaluation of the substrate oxidation at rest was performed by indirect calorimetry (Cortex Metalyzer 3B, Biophysik, Leipzig, Germany). Subjects were instructed to sleep approximately 8 h the night before, perform a 12-h fast, not exercise within 48 h prior to the evaluation, and not to consume caffeine or alcohol-containing beverages within 24 h prior to evaluation.

The indirect calorimetry procedure occurred between 7:00 am and 8:00 pm. The laboratory had low-light conditions, controlled noise levels, temperature between 23 and 25 °C, and relative humidity between 40 and 60%. The participants remained comfortable in a supine position for 30 min, remaining awake and immobile for the last 30 min.

#### Body composition

Body composition was measured by indirect dual energy radiological absorptiometry (DXA) (GE, Prodigy Advance model, GE Lunar software, Madison, USA). The percentages of total fat and fat per segment were quantified.

#### Lipid profile

After an overnight fast, 5-10 mL blood samples were collected from the antecubital vein of participants and transferred into tubes without anticoagulant. Blood samples were centrifuged at 3500 rpm for 15 min, at 4 °C, and the serum was separated into 1.5 mL aliquots and stored in a freezer at − 80 °C until analysis. Serum total cholesterol (TC), HDL cholesterol, LDL cholesterol, and triglyceride (TG) concentrations were determined using LABTEST kits (Lagoa Santa, Minas Gerais, Brazil), according to the manufacturer’s instructions. The analysis was carried out at the Laboratory of Research on Clinical Biochemistry of the UFRN, using the biochemical analyzer LABMAX PLENNO (Labtest, Lagoa Santa, Minas Gerais, Brazil).

#### Statistical analysis

The descriptive analysis was performed using percentage, mean, and standard deviation, according to the data type. The t-test was used to compare the pre-post intervention means (curcumin and placebo). To verify differences between pre-placebo and pre-curcumin, as well as post-placebo and post-curcumin, the paired t-test was used. Statistical analysis was performed using IBM SPSS Statistics v.22 and a significance level of 5% was adopted for all analyses.

## Results

Initially, 30 participants were enrolled, however 9 abandoned the protocol and 1 was excluded as he stopped using ART. Thus, 20 individuals completed the study. Overall, 12 (60%) were male and 8 (40%) female, with a mean age of 45.5 (9.7) years. The sample were heterogeneous with respect to HIV infection, demographic and lifestyle variables (Table 1).

**Table 1:** Clinical and socioeconomic aspects of people living with HIV/AIDS

Variables	Categories	Frequency (*n* = 20)	Percentage (%)
Time of HIV diagnosis	Up to 57 months 58 to 126 months 127 to 183 months 184 to 288 months	5 5 5 5	25 25 25 25
Time on Antiretroviral Therapy	Up to 57 months 58 to 120 months 121 to 180 months 181 to 240 months	5 6 5 4	25 30 25 20
Combination Antiretroviral Therapy	NRTI + NtRTI + NNRTI NRTI + NNRTI PI + NRTI + NtRTI PI + NRTI PI + NRTI + NNRTI NRTI + PI + II	7 6 2 3 1 1	35 30 10 15 5 5
Comorbidities	None Hypertension Depression Type 2 diabetes Type 2 diabetes, hypertension, depression, and osteoporosis	14 3 1 1 1	70 15 5 5 5
Work	Yes No	7 13	35 65
Alcohol consumption	Yes No	10 10	50 50
Smoke	Yes No	3 17	15 85
Schooling	Middle school High School College	4 12 4	20 60 20
Family income^a^	Up to 1 Brazilian salary Up to 2 Brazilian salary Up to 3 Brazilian salary Up to 4 Brazilian salary	6 6 4 4	32 3 5 26

*NRTI* Nucleoside Reverse Transcriptase Inhibitor, *NtRTI* nucleotide reverse transcriptase inhibitors, *NNRTI* Non-nucleoside reverse transcriptase inhibitors, *PI* Protease inhibitors, *II* Integrase Inhibitor

^a^Family income expressed in Brazilian salary (1 Brazilian salary corresponds approximately to US$ 260.00)

The participants’ food intake remained constant throughout the study period and both the EG and CG consumed more unprocessed or minimally processed foods (Table 2). No change was observed in the respiratory quotient or body composition of subjects after curcumin supplementation. However, TG levels increased after curcumin supplementation in the EG (Table 3). No side effects were observed during curcumin supplementation. There was no significant difference in the baseline values (pre) between EG and CG.

**Table 2:** Dietary intake of people living with HIV/AIDS

	Experimental Group (n = 20)		*P* value		Control Group (n = 20)		*P* value	
	Pre	Post	Curcumin Effect	Time Effect (Pre)	Pre	Post	Placebo Effect	Time Effect (Post)
24 h-Food Recall								
Energy (Kcal/day)	1837.1 (717.6)	1782.1 (739.3)	0.805	0.730	1736.5 (925.0)	1680.6 (580.4)	0.785	0.632
Protein (g/kg/day)	1.3 (0.6)	1.4 (0.7)	0.972	0.615	1.5 (0.7)	1.2 (0.7)	0,216	0.701
Protein (%)^a^	19.4 (5.6)	19.9 (6.1)	0.741	0.172	21.8 (5.5)	19.1 (6.8)	0.268	0.701
Carbohydrate (%)^a^	54.9 (12.1)	53.5 (11.2)	0.653	0.666	56.5 (9.2)	58.2 (8.3)	0.539	0.143
Lipid (%)^a^	25.7 (9.3)	26.6 (7.0)	0.692	0.139	21.7 (7.0)	22.7 (7.5)	0.602	0.100
Saturated Fat Acid (%)^a^	7.6 (4.2)	9.3 (2.9)	0.113	0.961	7.6 (3.3)	7.3 (3.4)	0.760	0.055
Cholesterol (g/day)	306.2 (146.5)	383.3 (290.6)	0.346	0.585	346.5 (293.0)	306.5 (205.0)	0.509	0.341
Fiber (g/day)	22.4 (15.3)	21.7 (8.6)	0.846	0.947	22.7 (12.0)	23.3 (9.4)	0.828	0.596
Food Consumption Markers								
Groups 1–5	55.0 (13.0)	56.3 (13.9)	0.663	0.897	55.7 (20.6)	54.6 (15.6)	0.761	0.715
Groups 6–10	18.9 (12.0)	14.6 (11.6)	0.188	0.349	15.4 (10.8)	16.4 (11.9)	0.731	0.620

All values are expressed as mean (standard deviation). ^a^Results in percentage of energy contribution

**Table 3:** Effect of curcumin supplementation on lipid profile, respiratory quotient, and body composition in people living with HIV/AIDS

	Experimental Group (*n* = 20)		*P* value		Control Group (*n* = 20)		*P* value	
	Pre	Post	Curcumin Effect	Time Effect (Pre)	Pre	Post	Placebo Effect	Time Effect (Post)
Total Cholesterol (mg/dL)	197.1 (39.9)	206.9 (52.4)	0.067	0.698	203.0 (55.2)	204.1 (46.1)	0.845	0.856
LDL (mg/dL)	124.6 (30.2)	126.1 (39.5)	0.781	0.681	120.9 (33.8)	128.6 (33.6)	0.318	0.814
HDL (mg/dL)	37.4 (11.9)	35.3 (13.8)	0.355	0.723	39.1 (17.6)	35.1 (13.2)	0.257	0.981
Triglycerides (mg/dL)	182.1 (131.9)	219.0 (140.3)	0.004*	0.737	195.9 (125.7)	198.9 (127.0)	0.799	0.638
Respiratory Quotient (RQ)	0.9 (0.1)	0.8 (0.1)	0.728	0.426	0.8 (0.0)	0.8 (0.1)	0.774	0.579
Total Body Fat (%)	26.9 (7.3)	26.8 (7.4)	0.785	0.973	29.3 (9.2)	28.8 (9.0)	0.035*	0.612
Arms Fat (%)	23.0 (8.7)	22.7 (9.2)	0.467	0.860	26.1 (11.3)	25.9 (11.6)	0.643	0.468
Legs Fat (%)	22.7 (8.7)	23.0 (9.1)	0.233	0.834	24.8 (10.2)	24.6 (9.8)	0.536	0.702
Trunk Fat (%)	31.1 (9.0)	30.2 (8.7)	0.338	0.781	33.6 (10.3)	32.7 (10.2)	0.019*	0.575
BMI (kg/m^2^)	23.7 (2.1)	23.7 (2.2)	0.832	0.934	25.0 (5.4)	25.0 (5.5)	0.653	0.426

All values are expressed as mean (standard deviation). *LDL* LDL-cholesterol, *HDL* HDL-cholesterol, *BMI* Body Mass Index. *Significant values (*P* < 0.05)

## Discussion

This study was the first clinical trial to investigate the influence of curcumin supplementation on metabolic and lipid parameters in people living with HIV/AIDS. Both experimental and control groups presented similar characteristics regarding dietary intake, body composition, substrate oxidation, and blood lipid profile at baseline. The main finding was the increase in serum TG levels after curcumin supplementation. None of the other parameters studied changed after the intervention.

Curcumin supplementation did not alter the energy substrate oxidation at rest or body composition of participants. Also, as shown in Table 3, the time effect was not significant, indicating that 30 days of curcumin supplementation was not sufficient to influence these parameters in the population studied. These findings may be associated with the complex mechanisms of HIV lipodystrophy. High carbohydrate oxidation at rest, also observed in other studies, is related to complex mechanisms such as mitochondrial dysfunction and impairment of fatty acid transport proteins [27, 28]. Substrate oxidation is influenced by central fat accumulation, a common feature of lipohypertrophy, which is hard to reverse, even with exercise or dietary interventions [29, 30]. Also, the precision error of the bone tissue can reach up to 2% and, in soft tissues, the standard error is based on the assumption of a constant hydration status of these tissues [31].

In the present study, the increase in serum TG levels after curcumin supplementation was contrary to previous studies which evaluated the effect of curcumin on the lipid profile of healthy individuals [32] and patients with chronic diseases, who have an inflammatory but not infectious profile, such as obesity [33], metabolic syndrome [34], and type 2 diabetes mellitus [35]. In these studies, the TG levels decreased after curcumin supplementation. Previous studies with people living with HIV/AIDS have shown only beneficial effects of curcumin supplementation on diarrhea [36], viral replication [37], and inflammation [38].

Only one clinical trial with elderly subjects found that curcumin supplementation did not significantly change serum TC, LDL, HDL, and TG levels over time. Interestingly, the authors observed that concentrations of plasma curcumin and serum cholesterol were positively correlated [39]. Some authors state that the curcumin effect in lowering TC and TG may be more efficient in cases of concomitant high fat diet [15]. Considering this fact, it is important to highlight that in the present study the participants met the daily recommendation for fat intake and none of them had a high fat diet.

However, it is possible that the increase in serum TG levels found in the experimental group of this study may have been partially influenced by diet. Although there is no gold standard method for assessing dietary intake, it is not a new fact that dietary composition influences serum lipids and lipoproteins [40]. Previous studies evaluating the effect of curcumin on the serum lipid profile [32–35] did not control food consumption. In the present study, in addition to the advice to maintain habitual eating habits, food consumption was also investigated. Although no significant differences in the dietary intake of the participants was observed, the high standard deviation of the results confers a certain variation in energy and macronutrient intake, which may have influenced the results regarding the lipid profile, especially serum TG levels. For example, the high intake of simple carbohydrates raises blood concentrations of TG. This phenomenon is known as carbohydrate-induced hypertriglyceridemia [41]. Moreover, individual genetic polymorphisms linked to the lipid metabolism, an aspect not investigated in the present study, are influenced by dietary intake and can modulate the lipid profile differently in people [42–44].

In addition, the participants of this study were physically active and previously enrolled in a 6-month exercise plan. This fact may have affected the effect of curcumin supplementation on the serum lipid profile, since physical exercise promotes several metabolic adaptations which contribute to preservation of lean mass and improvement in insulin sensitivity and lipid profile [45]. Perhaps curcumin supplementation may generate more positive results in the lipid profile of sedentary people living with HIV/AIDS.

The increase in serum TG levels after curcumin supplementation observed in our study also demonstrates the complexity of the metabolic alterations developed by patients with HIV under ART. These metabolic changes have a multifactorial etiology and their pathophysiological mechanisms are not fully understood. Feeney and Mallon [46] discussed the overall effects of ART on lipid profiles. The majority of antiretroviral drugs present dyslipidemic properties. Considering that all participants from both the experimental and control groups in this study were exposed to a combination of ART, we assume that the effect of the antiretroviral drugs used may have masked the potential benefits of the curcumin. The metabolic abnormalities related to ART found in HIV-infected patients are complex and encompass several metabolic pathways and mechanisms [47–50].

Clinical studies [32–35] and reviews [8, 15] have demonstrated the lipid-lowering effect of curcumin. However, this evidence is questionable in some meta-analyses [4, 51]. Apparently, this effect can be concealed by heterogeneous populations and a lack of methodological standardization [4]. In addition, the therapeutic efficacy of curcumin is still limited due to characteristics such as poor water solubility, low bioavailability, chemical instability, photodegradation, rapid metabolism, and a short half-life. Evidence about this bioactive compound has stimulated the development of new pharmaceutical formulas and new delivery systems which may improve the efficacy of curcumin [52].

Our study had some limitations. The immunological, virological, hormonal, and genetic aspects were not considered or controlled in this study. Besides that, our study had a limited number of participants. However, the cross over nature of the analysis allowed us to conclude the increasing of triglycerides found in this cohort is worthy of further investigation.

## Conclusion

Curcumin supplementation did not alter the energy substrate oxidation at rest, nor the body composition of physically active people living with HIV/AIDS under ART but did increase serum TG levels in this population. Further studies, with a larger sample cohort, different curcumin dosages, and longer intervention times are needed to validate current observations. In addition, the influence of physical activity, dietary intake, and genetic polymorphisms must be considered in future studies to better understand the impact of curcumin supplementation on the lipid profile of people living with HIV/AIDS, under antiretroviral therapy.

## Additional file

Additional file 1: Evaluation form. (PDF 231 kb)

## Data Availability

The datasets used are available from the corresponding author on reasonable request.

## Abbreviations

AIDS: Acquired immune deficiency syndrome
ART: Antiretroviral therapy
EG: Experimental group
CG: Control group
HDL: High-density lipoprotein
HIV: Human immunodeficiency virus
LDL: Low-density lipoprotein
TC: Total cholesterol
TG: Triglycerides
