# Supplementary material for: Influence of curcumin supplementation on metabolic and lipid parameters of people living with HIV/AIDS: a randomized controlled trial
Source: BMC Complement Altern Med. 2019 Aug 6;19:202. doi: 10.1186/s12906-019-2620-7 (PMC6685220; doi:10.1186/s12906-019-2620-7)
Supplement: Supplementary file 1 — Evaluation form. (PDF 231 kb) [file 12906_2019_2620_MOESM1_ESM.pdf]

Projeto VIVER

UFRN UNIVERSIDADE FEDERAL DO RIO GRANDE DO NORTE

FEDERAL UNIVERSITY OF RIO GRANDE DO NORTE
CENTER OF HEALTH SCIENCES
VIVER+ RESEARCH PROJECT

EVALUATION FORM

Anamnesis

Name: ______________________________________ ID Code: __________

Birth date / Age (years): _________________ Evaluation date: ___________

Diagnostic time (HIV/AIDS):______________ ART Therapy Time:__________

Current ART Combination: ____

For how long has it been in use? ____________________________________

Comorbidities ____

Other medications in use: ____

Work ( ) Yes ( ) No

Alcohol consumption ( ) Yes ( ) No

Smoke? ( ) Yes ( ) No

Education level: ( ) Middle school ( ) High School ( ) College

Family income: ____
